# Serum and follicular fluid organochlorine concentrations among women undergoing assisted reproduction technologies

**DOI:** 10.1186/1476-069X-8-32

**Published:** 2009-07-14

**Authors:** John D Meeker, Stacey A Missmer, Larisa Altshul, Allison F Vitonis, Louise Ryan, Daniel W Cramer, Russ Hauser

**Affiliations:** 1 Department of Environmental Health Sciences, University of Michigan, Ann Arbor, MI, USA; 2 Obstetrics, Gynecology and Reproductive Science, Brigham and Women's Hospital, Harvard Medical School, Boston, MA, USA; 3 Department of Environmental Health, Harvard School of Public Health, Boston, MA, USA; 4 Department of Biostatistics, Harvard School of Public Health, Boston, MA, USA; 5 Vincent Memorial Obstetrics and Gynecology Service, Andrology Laboratory and In Vitro Fertilization Unit, Massachusetts General Hospital, Boston, MA, USA

## Abstract

**Background:**

Exposure to persistent organic pollutants, including polychlorinated biphenyls (PCBs) and organochlorine pesticides, is widespread among the general population. There is evidence of adverse effects on reproduction and early pregnancy in relation to organochlorine exposure but human studies remain limited. The increased use of assisted reproductive technologies (ART) presents unique opportunities for the assessment of environmental influences on early pregnancy outcomes not otherwise observable in humans, but studies need to be designed to maximize the efficiency of the exposure data collected while minimizing exposure measurement error.

**Methods:**

The present study was conducted to assess the correlation between concentrations of organochlorines in serum and follicular fluid samples collected from a subset of women undergoing ART in a large study that took place between 1994 and 2003, as well as the temporal reliability of serum organochlorine concentrations among women undergoing multiple ART cycles in the study. PCB congeners (118, 138, 153, and 180), 1,1,1-trichloro-2,2-bis(*p*-chlorophenyl)ethane (p,p'-DDT), the DDT metabolite p,p'-DDE, hexachlorobenzene (HCB), oxychlordane, trans-nonachlor and mirex were measured in 72 follicular fluid samples and 265 serum samples collected from 110 women.

**Results:**

Organochlorine concentrations in paired serum and follicular fluid samples were correlated, with Pearson and Spearman coefficients ranging from 0.60 to 0.92. Serum organochlorine concentrations were two- to three-fold greater than in follicular fluid, and a significant inverse trend was observed in the distribution of follicular fluid:serum ratios with increasing molecular weight of the compound (p-value for trend < 0.0001). Serum organochlorine concentrations were highly reliable over the course of several months, with intraclass correlation coefficients ranging from 0.86 to 0.98. Finally, there was evidence for a declining trend in organochlorine concentrations between samples collected between years 1994–1998 and those collected in 1999–2003.

**Conclusion:**

Our results support the use of a single serum sample to adequately represent a more biologically relevant dose (concentrations in follicular fluid), as well as exposure levels over time, in epidemiological studies of ART outcomes in relation to organochlorine exposure.

## Background

There is concern for the potential adverse effects of environmental exposures to persistent organic chlorinated pollutants on fertility and pregnancy. Polychlorinated biphenyls (PCB) are a class of synthetic, persistent, lipophilic, chlorinated aromatic compounds that were widely used for decades in both industrial and consumer products. In the U.S., their production was banned in the late 1970's. However, as a result of their extensive use and persistence, PCBs remain ubiquitous environmental contaminants. The general population is exposed primarily through ingestion of contaminated foods (e.g., fish, meat, and dairy products), as PCBs bioaccumulate up the food chain. Like PCBs, the organochlorine pesticides such as 1,1,1-trichloro-2,2-bis(*p*-chlorophenyl)ethane (DDT), hexachlorobenzene (HCB), mirex, chlordane, and others are no longer used in the US and other industrialized nations, but exposures still occur through various routes and pathways due to their persistence in the environment. PCBs, organochlorine pesticides and their metabolites have long biological half-lives, and can be measured in the body months or years after an exposure has occurred [1]. Because of their persistence and ubiquity, measurable levels of serum PCBs and organochlorine pesticides or their metabolites are found in a high proportion of the general population [2]. Exposure to persistent organochlorines can result in an internal dose to the female reproductive tract, as these compounds have been measured in human follicular fluid [3,4], ovarian tissue [5], placenta, uterine muscle and amniotic fluid [6], as well as in embryos and fetuses [7,8], providing evidence of exposure to critical tissues during important windows of early development.

Organochlorine compounds have been associated with altered endocrine function, reduced fertility, and early pregnancy loss in experimental animals [9,10] and limited human studies [11-13], but more research is needed to understand potential risks associated with human exposure. An important limiting factor in assessing risk based on human studies is the degree of measurement error associated with the exposure estimate(s) used in a particular study. Two potential sources of exposure error in epidemiological studies, among others, include: 1) the ability of an exposure measure to predict an individual's biologically relevant dose to an agent (i.e. validity); and 2) the degree of temporal variability in exposure within and between individuals (i.e. reliability). If an exposure index is a poor surrogate for dose at the target organ of interest, and/or if there is large intra-individual variability in exposure over time compared to inter-individual variability, it will make it more difficult to detect an association between the exposure and outcome of interest through the dilution of effect estimates and loss of study power [14].

The present study was performed to determine how well blood levels of PCBs and organochlorine pesticides reflect levels at the target organ of interest, the ovary, and to quantify within-person variability in serum organochlorine concentrations over time. These results will be used to guide the design and analysis in our large ongoing epidemiological study on PCBs and organochlorine pesticide exposure and early pregnancy loss among women undergoing assisted reproduction technologies (ART) such as in vitro fertilization (IVF).

## Methods

### Study population

Details of the main study within which the present sub-study was performed has been previously described [15,16]. Briefly, between August 1994 and June 2003, couples undergoing IVF or intracytoplasmic sperm injection (ICSI) were recruited through three Boston area clinics to participate in a study of predictors of IVF outcome. The main study was conducted in two phases (1994 – 1998 and 1999 – 2003) corresponding with a five-year renewal of the study following the completion of the first five years. Study protocols were approved by the Human Research Committees at Brigham and Women's Hospital, Harvard School of Public Health, and the University of Michigan. Approximately 65% of couples who were approached agreed to participate in the study. Couples who required either donor oocytes or donor semen were excluded, as were couples who were gestational carriers and those who underwent gamete intra-fallopian transfer. Following these exclusions, 2,350 couples were enrolled in the main study. Many couples underwent multiple IVF/ICSI cycles (up to six), with the average being two cycles per couple.

### Collection of serum samples

Serum samples were collected from women in the study at "baseline" and at each IVF/ICSI cycle. Baseline samples were collected between days one and five of a menstrual cycle prior to the initiation of IVF/ICSI treatment. A serum sample was also collected at each cycle during the follicular phase immediately prior to human chorionic gonadotropin (HCG) administration. The serum fraction was separated for all blood samples by centrifugation for five minutes, aliquoted, and stored at -80C until analysis.

### Follicular fluid collection

Oocyte retrieval was performed approximately 36 hours after HCG treatment. The follicular fluid was obtained from the largest follicle (> 18 mm) visualized on ultrasound before using any flushing medium and only consisted of fluid from one follicle. This follicle was aspirated with the first puncture of the oocyte retrieval. The follicular fluid was transferred to a sterile Petri dish, and after the oocyte was removed, the fluid was placed into a 15 mL conical tube and centrifuged for 15 minutes. Within 30 minutes of collection, the supernatant was placed into a clean storage tube, aliquoted, and stored at -80C.

### Measurement of organochlorines in serum and follicular fluid

For the present exposure sub-study, serum samples from baseline and all repeated IVF/ICSI cycles among 110 women (a total of 265 serum samples) were analyzed for organochlorine concentrations. Women were selected randomly within strata based on age, year of enrollment, and IVF outcome as the first stage in a nested case-control analysis of organochlorines and early pregnancy loss. First-cycle follicular fluid samples were also analyzed for 62 of these women. An additional 10 follicular fluid samples from women not selected as part of the 110 above were also analyzed for organochlorines. These samples were included in the presentation of the population distribution of follicular fluid organochorine concentrations. Baseline serum samples were available for 85 (77%) of the women. The primary reason for missing baseline samples related to logistical challenges in collecting a sample from each woman in the study between days one and five of a menstrual cycle prior to treatment.

Measurement of organochlorines in serum and follicular fluid was conducted by the Organic Chemistry Analytical Laboratory, Harvard School of Public Health, and the method for serum has been described previously [17,18]. In brief, after liquid-liquid extraction and column chromatography clean-up the samples were analyzed by gas chromatography with dual Micro-ECD, on two capillary columns of different polarity using two internal standards. All final results were reported after subtracting the amount of the analyte measured in the procedural blank associated with the analytic batch. Samples were accompanied by the following quality control samples: a procedural blank, matrix spike samples, and a laboratory control sample. Each sample was spiked with two surrogate compounds to monitor the efficiency of the extraction procedure. Target analytes included 57 individual PCB congeners, p,p'-DDE, p,p'-DDT, HCB, oxychlordane, trans-nonachlor and Mirex. Since persistent organochlorines partition according to the lipid content of tissues, and serum lipid levels vary between fasting and non-fasting states, a correction for serum lipids is needed for the valid interpretation of serum levels [19]. Thus, levels were also reported after standardizing for serum lipids as units of ng/g total lipids to allow for comparison with other studies. Serum total cholesterol and triglycerides were measured enzymatically, and total lipids were calculated by Phillips formula [19,20]. However, lipids were only measured in 173 serum samples due to sample volume limitations.

The method for follicular fluid was similar to serum, and it was validated on anonymously collected follicular fluid samples prior to analyzing the study samples. Eight 5 g aliquots of pooled follicular fluid were fortified with 0.0144 ng of each target analyte per gram of fluid, and three aliquots were fortified with 0.0384 ng/g of follicular fluid. Percent recoveries for all target analytes, except oxychlordane, ranged from 80% to 104%. The mean (SD) recovery of oxychlordane was 40% (5.7%) because it was not completely eluted from the chromatography clean-up column since a non-polar solvent was used for all target analytes. Values were not recovery adjusted prior to reporting or statistical analysis.

Method detection limits (MDL) were determined as recommended by the US Environmental Protection Agency [21]. In serum the MDL values for all target analytes were < 0.05 ng/g, with most of the analytes < 0.01 ng/g. The MDL for *p,p'*-DDE was higher because unfortified serum had high *p,p'*-DDE concentrations at 6.3 ng/g. In follicular fluid the MDLs for all target analytes were < 0.005 ng/g with most of the analytes < 0.001 ng/g. The MDL for *p,p'*-DDE was higher because unfortified follicular fluid had high *p,p'*-DDE concentrations at 0.221 ng/g. Only results involving analytes with detectable concentrations in 100% of samples are presented to avoid issues of censorship or bias when including samples below the MDL.

### Statistical analysis

Data analysis was conducted using SAS software (version 9.1; SAS Institute Inc., Cary, NC). Distributions of organochlorine concentrations in follicular fluid and serum were tabulated and compared. Spearman rank correlations between analytes were then calculated within each matrix (follicular fluid and serum), as were Pearson correlation coefficients following the transformation of organochlorine concentrations by the natural logarithm (ln). Spearman and Pearson correlations were also calculated to assess the level of agreement between paired first-cycle follicular fluid and serum organochlorine concentrations. The ratio of organochlorine concentrations in follicular fluid to serum was calculated for all women and for each analyte. Ratio distributions were tabulated, and differences between analytes compared using linear regression with the various analytes ranked and assigned indicator variables based on chemical properties (i.e. molecular weight or number of chlorine atoms).

To assess between- and within-person variability in serum organochlorine concentrations, intraclass correlation coefficients (ICC) and their 95% confidence intervals were calculated for both wet-weight and lipid-standardized ln-adjusted concentrations of each analyte using SAS PROC MIXED [22]. ICC is a measure of the reliability of repeated measures over time, defined as the ratio of between-subject variance to total variance. ICC ranges from zero to one, with values near zero indicating poor reliability and values near one indicating high reliability [23]. Finally, to assess temporal patterns in organochlorine concentrations among women in our study, we compared serum organochlorine concentrations between the first and second phases of the main study using a student's t-test. We also fit a mixed effects model for each analyte, where we calculated the fixed effect for IVF/ICSI cycle number, while accounting for random subject effects, to assess changes in serum organochlorine concentrations within individuals over time. This analysis was conducted using both wet-weight and lipid-standardized concentrations, as well as when using wet-weight concentrations and including lipids as a covariate.

## Results

The mean (SD) age among the women was 36 (4.0) years, and ranged from 25 to 44 years. Among the 265 serum samples, 14 women contributed a single sample, 57 women contributed two samples, 25 women contributed three samples, eight women contributed four samples, five women contributed five samples, and one woman contributed six samples. The calendar time between the collection of the first and the final serum sample ranged from two to 24 months, with a median of five months. The distribution of organochlorine concentrations in first cycle serum and follicular fluid samples are presented in Table 1. In both serum and follicular fluid, *p,p'*-DDE and PCB 153 had the highest concentrations among the pesticides and PCB congeners, respectively. Correlation coefficients between compounds in both serum and follicular fluid are presented in Table 2. Organochlorine pesticides (i.e., p,p'-DDE, p,p'-DDT, HCB, oxychlordane, trans-nonachlor and mirex) were moderately correlated with each other and with PCB congeners (correlation coefficients [r] ranged from 0.3 and 0.7) with a couple of exceptions; in follicular fluid trans-nonachlor was strongly correlated with oxychlordane and with individual PCB congeners (r ≥ 0.7), and in both matrices weak correlations were found for mirex with HCB and p,p'-DDE (r ≤ 0.2). As expected, concentrations of PCB congeners were strongly correlated with one another in both serum and follicular fluid (r between 0.7 and 0.9).

**Table 1:** Distribution of organochlorine pesticide and PCB concentrations (ng/g wet weight) in all first cycle serum (n = 110) and follicular fluid (n = 72) samples

	Geometric Mean	Selected Percentiles						
		
		10^th^	25^th^	50^th^	75^th^	90^th^	95^th^	Max.
HCB								
Serum	0.093	0.051	0.069	0.090	0.114	0.151	0.195	2.31
FF	0.032	0.017	0.025	0.035	0.044	0.057	0.072	0.588

Oxychlordane								
Serum	0.045	0.024	0.033	0.046	0.062	0.088	0.094	0.156
FF	0.011	0.006	0.008	0.012	0.014	0.018	0.023	0.036

Trans-nonachlor								
Serum	0.090	0.045	0.068	0.092	0.128	0.168	0.205	0.485
FF	0.018	0.008	0.013	0.020	0.027	0.031	0.040	0.138

Mirex								
Serum	0.014	0.006	0.010	0.014	0.022	0.031	0.051	0.100
FF	0.004	0.001	0.002	0.004	0.006	0.008	0.009	0.040

p,p'-DDE								
Serum	1.23	0.430	0.700	1.08	1.81	3.93	8.75	24.2
FF	0.384	0.123	0.223	0.363	0.878	1.85	4.26	6.74

p,p'-DDT								
Serum	0.065	0.030	0.040	0.063	0.086	0.119	0.346	1.98
FF	0.013	0.005	0.009	0.014	0.019	0.028	0.047	0.116

PCB 118								
Serum	0.091	0.041	0.056	0.088	0.145	0.205	0.320	0.540
FF	0.028	0.010	0.017	0.032	0.047	0.073	0.096	0.132

PCB 138								
Serum	0.149	0.073	0.103	0.153	0.206	0.295	0.313	1.11
FF	0.043	0.019	0.034	0.047	0.071	0.100	0.139	0.380

PCB 153								
Serum	0.257	0.119	0.181	0.260	0.358	0.521	0.591	2.33
FF	0.064	0.024	0.047	0.072	0.110	0.148	0.211	0.778

PCB 180								
Serum	0.159	0.073	0.119	0.168	0.229	0.293	0.350	1.98
FF	0.039	0.013	0.021	0.045	0.060	0.100	0.111	0.640

ΣPCB								
Serum	1.41	0.754	1.06	1.40	1.84	2.49	3.01	10.3
FF	0.350	0.127	0.252	0.384	0.534	0.709	0.970	3.23

**Table 2:** Spearman correlation coefficients between organochlorine concentrations (ng/g) in follicular fluid (n = 72) and serum (n = 110)

	HCB	Oxy-chlordane	Trans-nonachlor	Mirex	p,p-DDE	p,p-DDT	PCB 118	PCB 138	PCB 153	PCB 180	ΣPCB
HCB		0.47	0.44	0.13	0.50	0.56	0.43	0.41	0.41	0.35	0.43
Oxychlordane	0.50		0.87	0.54	0.33	0.53	0.70	0.66	0.71	0.68	0.73
Trans-nonachlor	0.42	0.88		0.61	0.43	0.54	0.76	0.69	0.75	0.70	0.77
Mirex	0.09	0.46	0.51		0.24	0.50	0.41	0.51	0.60	0.63	0.59
p,p'-DDE	0.60	0.42	0.43	0.11		0.69	0.43	0.59	0.56	0.41	0.52
p,p'-DDT	0.60	0.57	0.63	0.32	0.72		0.52	0.61	0.64	0.58	0.64
PCB 118	0.38	0.59	0.62	0.33	0.35	0.56		0.78	0.80	0.68	0.86
PCB 138	0.52	0.62	0.62	0.37	0.55	0.65	0.79		0.95	0.81	0.93
PCB 153	0.52	0.61	0.62	0.41	0.47	0.63	0.79	0.94		0.92	0.97
PCB 180	0.43	0.58	0.60	0.45	0.33	0.56	0.70	0.80	0.93		0.90
ΣPCB	0.48	0.63	0.62	0.42	0.40	0.60	0.82	0.89	0.95	0.91	

*Correlation coefficients above diagonal are for follicular fluid, below diagonal are for serum.

**Figure 1:**
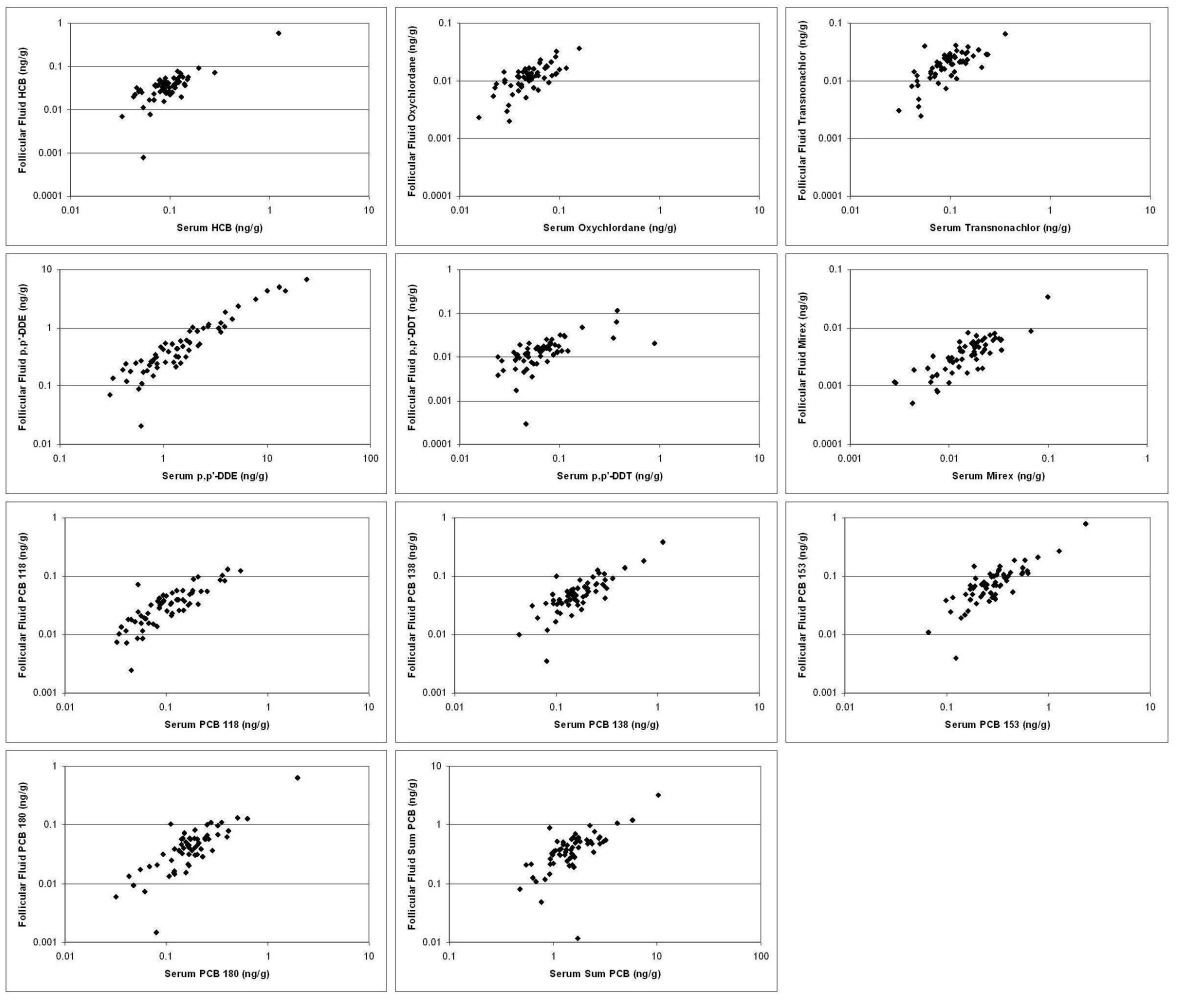
**Scatterplots of organochlorine concentrations measured in first-cycle serum and follicular fluid (n = 62)**. Spearman correlation coefficients between media were as follows (all p-values < 0.05): HCB, 0.66; oxychlordane, 0.66; transnonachlor, 0.65; p,p'-DDE, 0.92; p,p'-DDT, 0.74; mirex, 0.80; PCB 118, 0.82; PCB 138, 0.74; PCB 153, 0.73; PCB 180, 0.73; Sum PCB, 0.68.

Plots comparing organochlorine concentrations in first cycle follicular fluid and serum are presented in Figure 1. All correlation coefficients were significantly greater than zero (p < 0.05). With the exception of the sum of PCBs, at least one of the two correlation coefficients presented (the Pearson or Spearman) would be classified as strong (≥ 0.7) for all analytes. Correlation coefficients for the sum of PCBs were just below 0.7, while the strongest correlation (Spearman r > 0.9) was for p,p'-DDE.

The organochlorine concentrations in follicular fluid were consistently lower than levels in serum. The distributions of the ratios of follicular fluid to serum organochlorine concentrations are presented in Table 3. Despite the strong correlations between follicular fluid and serum organochlorine concentrations, there was considerable between-person variability in these ratios for most of the analytes. For instance, ratios for PCB 118 ranged from 0.06 to 1.38. Follicular fluid:serum ratios were not associated with age or BMI (data not shown). One woman had high ratios that would be considered an outlier (> 3 SD from the mean) for several analytes, with one ratio (PCB 118) exceeding 1.0 and a number of other ratios just below 1.0. There was also a significant inverse trend between mean follicular fluid:serum ratio and molecular weight in a linear regression model that used an indicator variable to rank analytes according to molecular weight (p < 0.0001). That is, as molecular weight increased, the follicular fluid:serum ratio decreased. For example, the median (25^th^, 75^th^) ratio value for the compound with the lowest molecular weight (HCB, MW = 285) was 0.37 (0.29, 0.49) compared to 0.22 (0.17, 0.30) for the compound with the highest molecular weight (mirex, MW = 546). A statistically significant inverse trend was also found when assigning an indicator variable to rank analytes according to the number of chlorine atoms, though it was not as strong as the trend observed for molecular weight (data not shown).

**Table 3:** Distribution of follicular fluid:serum ratios with analytes listed in ascending order by molecular weight (N = 62)

	Molecular weight	Mean	Selected Percentiles							
			
			Min.	10^th^	25^th^	50^th^	75^th^	90^th^	95^th^	Max.
HCB	285	0.39	0.13	0.22	0.29	0.37	0.49	0.55	0.60	0.69
p,p'-DDE	318	0.32	0.03	0.18	0.24	0.32	0.41	0.46	0.49	0.55
PCB 118	326	0.32	0.06	0.18	0.22	0.30	0.39	0.46	0.48	1.38*
p,p'-DDT	355	0.22	0.02	0.11	0.14	0.22	0.27	0.32	0.36	0.47
PCB 138	361	0.31	0.04	0.17	0.24	0.30	0.37	0.43	0.51	0.99*
PCB 153	361	0.27	0.03	0.14	0.20	0.25	0.34	0.39	0.41	0.79*
PCB 180	395	0.25	0.02	0.12	0.18	0.25	0.31	0.40	0.41	0.94*
Oxychlordane	426	0.24	0.06	0.12	0.17	0.24	0.29	0.35	0.37	0.52
Trans-nonachlor	444	0.21	0.05	0.10	0.16	0.20	0.25	0.29	0.32	0.71*
Mirex	546	0.24	0.06	0.11	0.17	0.22	0.30	0.39	0.42	0.54
										
ΣPCB		0.27	0.06	0.14	0.19	0.26	0.33	0.38	0.42	0.97*

*One woman was consistently an outlier for high ff:serum ratio for PCBs and trans-nonachlor. This woman also had the highest ratio for oxychlordane.

The ICC values presented in Table 4, all of which are near 1.0, demonstrate the high level of reliability for serum organochlorine concentrations over time. ICC values ranged from 0.85 for oxychlordane to 0.98 for p,p'-DDE. Finally, Table 5 provides evidence for a decline in organochlorine levels among women in the study over time, as concentrations in first cycle follicular fluid and serum were consistently higher among women in the first phase of the study (1994–1998) compared to the second phase (1999–2003). In linear mixed effects models, cycle was inversely associated with wet weight serum concentrations of mirex and PCB congeners 138, 153 and 180. There were suggestive inverse associations between cycle number and p,p'-DDE and p,p'-DDT (p-values = 0.07 and 0.06, respectively). However, these associations were no longer statistically significant when concentrations were lipid-standardized (n = 173, results not shown).

**Table 4:** Intraclass correlation coefficients (95% confidence intervals) for organochlorine concentrations (ng/g) in serum

	Wet-weight concentrations		Lipid-standardized	
	All samples; n = 266	Excluding baseline; n = 178	All samples; n = 173	Excluding baseline; n = 105

HCB	0.95 (0.93, 0.96)	0.97 (0.95, 0.98)	0.95 (0.92, 0.97)	0.97 (0.95, 0.98)
Oxychlordane	0.85 (0.80, 0.90)	0.91 (0.86, 0.94)	0.90 (0.85, 0.93)	0.93 (0.88, 0.96)
Transnonachlor	0.88 (0.83, 0.91)	0.94 (0.92, 0.96)	0.95 (0.92, 0.97)	0.94 (0.90, 0.97)
Mirex	0.88 (0.83, 0.91)	0.95 (0.93, 0.97)	0.90 (0.85, 0.93)	0.95 (0.92, 0.97)
p,p'-DDE	0.98 (0.97, 0.99)	0.98 (0.97, 0.99)	0.98 (0.97, 0.99)	0.98 (0.97, 0.99)
p,p'-DDT	0.93 (0.91, 0.95)	0.96 (0.95, 0.98)	0.95 (0.92, 0.97)	0.96 (0.92, 0.98)
PCB 118	0.92 (0.89, 0.94)	0.97 (0.96, 0.98)	0.97 (0.95, 0.98)	0.97 (0.95, 0.99)
PCB 138	0.91 (0.87, 0.93)	0.96 (0.94, 0.97)	0.96 (0.94, 0.98)	0.97 (0.95, 0.98)
PCB 153	0.91 (0.88, 0.94)	0.96 (0.93, 0.97)	0.97 (0.95, 0.98)	0.97 (0.94, 0.98)
PCB 180	0.91 (0.87, 0.93)	0.95 (0.93, 0.97)	0.96 (0.94, 0.97)	0.97 (0.95, 0.98)
Sum PCB	0.83 (0.77, 0.88)	0.92 (0.89, 0.95)	0.88 (0.83, 0.92)	0.93 (0.88, 0.96)

Cholesterol^b^	0.58 (0.43, 0.71)	0.67 (0.49, 0.82)		
Triglycerides^b^	0.85 (0.78, 0.90)	0.86 (0.77, 0.92)		
Total lipids^b^	0.78 (0.69, 0.86)	0.83 (0.71, 0.90)		

n = 265 serum samples from 110 women.

^a^values transformed by the natural logarithm.

^b^cholesterol, triglycerides, and total lipids only available for 173 samples.

**Table 5:** Geometric mean organochlorine concentrations in first cycle follicular fluid or serum (ng/g wet weight) by study period

	Follicular fluid			Serum		
	Phase 1 (1994–1998); n = 31	Phase 2 (1999–2003); n = 41	p-value	Phase 1 (1994–1998); n = 56	Phase 2 (1999–2003); n = 54	p-value

HCB	0.036	0.029	0.2	0.104	0.083	0.05
Oxychlordane	0.013	0.010	0.02	0.052	0.039	0.003
Trans-nonachlor	0.021	0.016	0.09	0.106	0.075	0.001
Mirex	0.004	0.003	0.10	0.015	0.013	0.2
p,p'-DDE	0.48	0.32	0.2	1.53	0.98	0.01
p,p'-DDT	0.017	0.010	0.02	0.086	0.049	<0.0001
PCB 118	0.034	0.025	0.08	0.106	0.079	0.02
PCB 138	0.056	0.035	0.04	0.181	0.122	0.0004
PCB 153	0.086	0.052	0.02	0.306	0.214	0.0002
PCB 180	0.051	0.032	0.01	0.187	0.134	0.005
Sum PCB	0.47	0.28	0.004	1.57	1.26	0.03

^a^t-test using ln-transformed values.

## Discussion

In the present study, we found a strong correlation between the concentrations of organochlorine pesticides and PCBs measured in paired serum and follicular fluid samples, and a high degree of reliability in serum organochlorine concentrations within an individual over time periods of less than two years. Thus, measuring organochlorine concentrations in a single serum sample likely reflects concentrations in follicular fluid over the course of repeated IVF/ICSI cycles. The reliability of serum organochlorine levels has been assessed throughout different stages of pregnancy and early development [24-28]. However, to our knowledge this is the first study to assess reliability of serum organochlorine concentrations over time among women attempting to become pregnant.

In studies of patients undergoing assisted reproduction technologies, organochlorine levels measured in follicular fluid are likely to be a biologically relevant marker of exposure since it reflects the ovarian microenvironment and thus organochlorine exposure of the maturing oocyte. During IVF treatment, women undergo superovulation to maximize the number of oocytes that can be retrieved. During the pre-antral phase, follicular fluid, a thecal blood filtrate, accumulates and suspends the oocyte and the cumulus cells surrounding it. Just prior to ovulation, when the majority of a follicle's volume consists of follicular fluid, the basement membrane becomes increasingly permeable and eventually breaks down [29]. When the follicles are collected the follicular fluid is aspirated and usually discarded. However, in addition to studies among patients undergoing IVF/ICSI allowing for the assessment of early pregnancy outcomes not observable in women conceiving naturally (e.g. implantation, embryo quality, etc.), another innovative aspect is the ability to collect and analyze the follicular fluid for environmental contaminants as a biologically relevant exposure biomarker. However, serum may remain a more desirable matrix in epidemiological studies since: 1) the analytical methods for the measurement of organochlorines in serum are well-developed; 2) organochlorine concentrations tend to be higher in serum which would increase sample detection rates in research studies; 3) many follicle aspiration protocols involve the use of a flushing medium (though one was not used in the present study) which would introduce error in follicular fluid organochlorine concentrations; and 4) most studies to date have measured organochlorine concentrations in serum, which facilitates straight-forward comparisons of exposure between studies in human risk assessments – especially in the case of reproductive health studies among the general population in which the collection of follicular fluid is not possible.

Several previous studies have measured organochlorine concentrations in follicular fluid [3,4,30-34]. However, most studies were small in size and only two of the studies reported on the association between organochlorine concentrations in follicular fluid and serum. The first, a Belgian study among 8 women, reported strong correlations (>0.7) between follicular fluid and serum concentrations of PCBs 138, 153 and 180, but not PCB 118. The second study, which was conducted among 74 women from three regions of Canada, reported moderate to weak correlations for DDE (r = 0.53), oxychlordane (r = 0.23), HCB (r = 0.32), and sum of PCBs (r = 0.37) [30]. Reasons for the discrepancies in the level of agreement between matrices in the Canadian study and the present study are unclear, but may be due to differences in exposure levels among the two populations, differences in IVF treatment protocols and follicular fluid collection, or perhaps differences in analytical sensitivity since there was a high percentage (over 50% for some analytes) of non-detect samples in the Canadian study. A couple of the remaining studies stated that there were strong correlations between serum and follicular fluid organochlorine concentrations but did not present any quantitative measures [3,31].

The present study is the first to conduct an in-depth assessment of follicular fluid to serum organochlorine ratios. Baukloh et al. [33] reported higher PCB concentrations, but lower HCB concentrations, in follicular fluid compared to matched serum samples from 12 Austrian women. Conversely, in findings that were consistent with the present study, Pauwels et al. [34] reported serum PCB concentrations were on average approximately 3-fold higher in serum compared to follicular fluid in a study of eight Belgian women. The authors attributed this difference to the difference in lipid content between the two matrices – follicular fluid has been reported to be approximately 0.03% lipids [4] as compared to serum, which averaged nearly 0.6% lipids among women in the present study. Likewise, a Canadian study among 21 women reported serum concentrations of DDE and PCBs were approximately 2-fold higher than follicular fluid concentrations [3]. In the present study, we found that follicular fluid to serum organochlorine ratios were inversely associated with molecular weight, where HCB, which has the lowest molecular weight among the organochlorines measured, had the highest follicular fluid:serum ratios. This finding may be a result of the relative permeability of the basement membrane to the various organochlorine compounds, where lower molecular weight compounds may pass through to the follicle more easily as has been reported for serum proteins [35-37]. However, more research is needed to explain this trend for organochlorines and other environmental conditions. In addition, more information is needed on predictors of between-individual variability in follicular fluid:serum ratios, as higher ratios may represent individuals with increased susceptibility to organochlorine exposures in relation to fertility and early pregnancy since a greater proportion of a given exposure level may reach the target organ in those individuals. Alternatively, inter-individual differences may reflect variability due to treatment if they were somehow associated with the movement of chemicals into the follicle. For example, the influence of differing IVF treatment protocols, variable estrogen environments, and follicle size [38] on follicular fluid organochlorine concentrations and follicular fluid:serum ratios should be explored. Finally, data is also needed to determine whether ratios are variable over time within individuals.

Our finding of lower organochlorine concentrations in phase 2 of the study (1999–2003) compared to phase 1 (1994–1998) is consistent with other reports of temporal declines in exposure [39-42]. The distribution of serum organochlorine concentrations in the present study were similar to those presented among a subset of NHANES participants surveyed in 2001–2001 from the Third National Report on Human Exposure [2]. Among analytes detected in at least 50% of samples in the Third Report, geometric mean concentrations from first cycle serum samples in the present study were slightly higher compared to the female strata in the Third Report for PCB 153 (0.28 ng/g serum versus 0.16 ng/g) and PCB 180 (0.17 ng/g versus 0.11 ng/g), whereas p,p'-DDE concentrations were somewhat lower in the present study (1.44 ng/g compared to the 1.85 ng/g in the Third Report). Geometric mean serum concentrations of PCB 138, oxychlordane, and trans-nonachlor were also slightly lower in the present study.

## Conclusion

In conclusion, although the present study found evidence for declining organochlorine concentrations over time and between-individual and between-analyte variability in the ratio of follicular fluid to serum organochlorine concentrations, our results suggest that a single serum value indicates an individual's relative rank for biologically relevant dose in the ovary, as well as exposure level over time, in studies of ART outcomes in relation to organochlorine exposure.

## Abbreviations

ART: assisted reproductive technologies; BMI: body mass index; CDC: US Centers for Disease Control and Prevention; DDE: 1,1-dichloro-2,2-bis(p-chlorophenyl)ethylene; DDT: 1,1,1-trichloro-2,2-bis(p-chlorophenyl)ethane; EPA: US Environmental Protection Agency; HCB: hexachlorobenzene; HCG: human chorionic gonadotropin; ICC: intraclass correlation coefficient; ICSI: intracytoplasmic sperm injection; IVF: in vitro fertilization; MDL: method detection limit; MW: molecular weight; OC: organochlorine; PCB: polychlorinated biphenyl; SD: standard deviation.

## Competing interests

The authors declare that they have no competing interests.

## Authors' contributions

JDM: Data analysis, interpretation, manuscript composition; SAM: Biospecimen archive management, data interpretation, manuscript composition; LA: Analytical chemistry method development, sample analysis and interpretation; AFV: Biospecimen management, data management and analysis; LR: Statistical direction and interpretation; DWC: Cohort development, recruitment, and data collection; RH: Data interpretation and manuscript composition.
